# Annexin II as a Dengue Virus Serotype 2 Interacting Protein Mediating Virus Interaction on Vero Cells

**DOI:** 10.3390/v11040335

**Published:** 2019-04-09

**Authors:** Isah Abubakar Aliyu, King-Hwa Ling, Nur Fariesha Md Hashim, Jia-Yong Lam, Hui-Yee Chee

**Affiliations:** 1 Department of Medical Microbiology and Parasitology, Faculty of Medicine and Health Science, Universiti Putra Malaysia, 43400 UPM Serdang, Selangor, Malaysia; isahaa97@gmail.com (I.A.A); jiayonglam@gmail.com (J.-Y.L.); 2 Department of Medical Laboratory Science, Faculty of Allied Health Science, College of Health Science, Bayero University Kano, PMB 3011 Kano State, Nigeria; 3 NeuroBiology & Genetics Group, Department of Biomedical Sciences, Faculty of Medicine and Health Sciences, 43400 UPM Serdang, Selangor, Malaysia; lkh@upm.edu.my; 4 Genetics and Regenerative Medicine Research Centre, Faculty of Medicine and Health Sciences, 43400 UPM Serdang, Selangor, Malaysia; 5 Department of Biomedical Sciences, Faculty of Medicine and Health Sciences, 43400 UPM Serdang, Selangor, Malaysia; nurfariesha@upm.edu.my

**Keywords:** dengue, annexin II, Vero, siRNA, antibody-mediated inhibition

## Abstract

Recent evidence has demonstrated that dengue virus requires active filopodia formation for a successful infection. However, the cellular factor involved in the interaction has not been fully elucidated. We used a combination of virus overlay protein binding assay and LC-MS/MS, and identified annexin II as a dengue virus serotype 2 (DENV2) interacting protein on Vero cells, upon filopodia induction. Flow cytometry analysis showed annexin II on the Vero cells surface increased when DENV2 was added. The amount of annexin II in the plasma membrane fraction was reduced as the infection progressed. Antibody-mediated inhibition of infection and siRNA-mediated knockdown of annexin II expression significantly reduced DENV2 infection and production levels. Collectively, we demonstrated that annexin II is one of the host factor involved in DENV2 binding on Vero cells.

## 1. Introduction

Dengue virus is the most prevalent mosquito-borne virus, with an immense global health significance. It is an etiological agent of dengue fever, dengue hemorrhagic fever, and dengue shock syndrome. Dengue fever is endemic in more than 100 countries, and the disease is ubiquitous in tropical and subtropical countries, particularly in Asia and Latin America [1]. It is estimated that more than 2.5 billion people (40% of the world’s population) are at a risk of dengue infection [2].

Despite the increasing incidence of dengue virus infections, there are no specific antiviral agents or widely-accepted licensed vaccines available for treatment or prevention of a dengue virus infection. While research and development of dengue virus vaccines has been hampered by many factors, including antibody-dependent enhancement of infection [3], the original antigenic sin (“Hoskins effect”) hypothesis [4], and poor immunogenicity of subunit vaccines [5,6,7], antiviral drug discovery has begun to explore the possibility of developing host-oriented molecules acting on cellular functions that are essential for viruses to enter cells and replicate [8]. Identification of virus entry-associated proteins on the surface of target cells is important basic information for designing prevention and treatment strategies for viral diseases. Such therapeutic strategies have demonstrated promising results, as exemplified by the CCR5 receptor antagonist, maraviroc (“Selzentry”), which has been approved for clinical use in human immunodeficiency virus (HIV) infection [9]. Hence, a similar therapeutic strategy could be adapted for targeting a dengue virus host binding molecules, to prevent viral attachment and internalization. Many studies have been carried out to identify molecules involved in dengue virus–host interactions. Independent studies have shown that different proteins, including high-affinity laminin receptor [10,11], CD14-associated protein [12], and other, uncharacterized, proteins [13] might be involved in dengue virus–host cell interactions. However, it has been reported that most of these molecules are not involved in dengue virus high-affinity interaction or internalization [14].

Viruses have been shown to induce cytoskeletal rearrangements, leading to the formation of filopodia, which are crucial for virus infections [15] and internalization [16,17]. Viruses have been shown to utilize different signaling pathways, to activate filopodia for a successful infection. It has been reported that Epstein–Barr viruses and herpes simplex virus can activate Cdc42 small GTPases and Rac1 signaling pathways, to induce lamellipodia and filopodia formation in human fibroblast cells and epithelial cells lines [18]. Similarly, dengue virus 2 was reported to induce filopodia formation for successful infection in HMEC-1 cell, by the activation of Rac1 signaling pathway [19]. In addition, dengue virus particle was also ultrastructurally observed, in association with filopodia, in our earlier work [20], hence, this study aimed to identify molecules expressed upon filopodia induction and which are involved in dengue virus–host interactions, leading to an infection.

## 2. Materials and Methods

### 2.1. Induction of Filopodia Formation

Vero cells (ATCC^®^ CCL-81™) at 50–60% confluency were incubated with serum-free EMEM (Biowest, Nuaillé, France) for 24 h. The cells were either exposed to DENV2 (a clinical isolate from patients admitted to a local hospital) at a multiplicity of infection (MOI) of 5, or mock-exposed and incubated at 37 °C, with 5% CO_2_ for 30 min. Cells were then examined for filopodia formation, under an inverted light microscope (Olympus, Tokyo, Japan).

### 2.2. Virus Overlay Protein Binding Assay (VOPBA) and Protein Identification

Filopodia formation was chemically induced with bradykinin (Sigma-Aldrich, St. Louis, MO, USA) dissolved in DMSO. Vero cells in EMEM were treated with 200 ng of bradykinin or without bradykinin as control (mock-treated), and incubated at 37 °C with 5% CO_2_, for 30 min. The cells were examined under an inverted light microscope, as above.

Approximately 50 μg of plasma membrane protein was extracted from the filopodia-induced and mock-induced cells, using a Biovision plasma membrane protein extraction kit (BioVision, Milpitas, CA, USA) and were resolved by 12% SDS-PAGE. The gels were either stained with Bio-safe Coomassie Brilliant Blue G-250 (Bio-Rad, Hercules, CA, USA), or transferred onto polyvinylidene difluoride (PVDF) membranes (Millipore, Burlington, MA, USA), using a trans-blot semi-dry transfer system (Bio-Rad, USA). The membrane was stained with Ponceau S (Sigma-Aldrich, USA), for one hour, at room temperature, with gentle rocking, to verify protein transfer. It was then blocked in a blocking buffer (TBS + 5% skim milk) at room temperature, for two hours, and washed three times with TBS-T (TBS containing 0.05% Tween 20), for ten minutes each. The membrane containing transferred protein was either incubated with partially purified DENV2 or C6/36 cell lysate, prepared according to Chee and AbuBakar [21], overnight, at 4 °C. The membrane was vigorously washed three times with TBS-T, for ten minutes each, and incubated with 1:1000 dilution of mouse anti-DENV2 E protein antibody (Abcam, Cambridge, UK) in 3% skimmed milk, overnight, at 4 °C. The membrane was washed again three times with TBS-T, for ten minutes each. The membrane was then incubated with 1:7000 dilution of a goat anti-mouse HRP-conjugated secondary antibody (BioLegend, San Diego, CA, USA), in 3% skimmed milk, at room temperature, for two hours. It was washed again, three times, with TBS-T and was developed with electrochemiluminescence (ECL) (Bio-Rad, USA). Images were acquired using the Syngene gel/chemiluminescence (gel/chemi) documentation system (Syngene, Cambridge, UK). For protein identification, the corresponding DENV2 binding band from parallel Coomassie blue-stained gels was excised and sent to Proteomics International (Australia) for protein identification, using LC-MS/MS. The sample was processed, according to Bringans et al. [22]. The generated spectra were analyzed by Mascot sequence matching software (Matrix Science, 2.2.04, London, UK). Protein concentration was determined by a Bradford assay using a Nanodrop 1000 spectrophotometer (Thermo Fisher Scientific, Waltham, MA, USA).

### 2.3. Flow Cytometry Analysis of Annexin II upon Dengue Virus Serotype 2 Exposure

Vero cells were scraped and collected by centrifugation at 200× *g* for 5 min, followed by resuspension in serum-free EMEM. Subsequently, 1 × 10^5^ Vero cells were exposed to DENV2 or DENV2 which was inactivated at 56 °C, for 45 min [23], at a MOI of 2, or mock-exposed, at 37 °C with 5% CO_2_ for 30 min. The cells were then washed with FACS buffer (1% FBS, 0.1% NaN3, in PBS), fixed with 1% paraformaldehyde in PBS, for 20 min, followed by blocking with blocking buffer (3% BSA in PBS) for 30 min and then incubated with 1:10 dilution of rabbit polyclonal anti-annexin II antibody (ab41803, Abcam, UK) or isotype control antibody (rabbit polyclonal anti-LAT, sc-7948, Santa Cruz, CA, USA) for 1 h on ice. The cells were then washed with an FACS buffer, for three times, centrifuged at 300× *g* for 5 min. Thereafter, the cells were resuspended in 1:100 dilution of Alexa Fluor^®^ 488-conjugated donkey anti-rabbit IgG (Thermo Fisher Scientific, USA) for 1 h on ice, washed twice with FACS buffer. Following staining, the cells were resuspended in PBS, and analyzed, using BD FACS Canto II flow cytometer and BD FACSDiva analysis software (BD Biosciences, San Jose, CA, USA). Unstained Vero cells was used as control.

### 2.4. Western Blot Analysis of Annexin II upon Dengue Virus Serotype 2 Infection

Vero cells were either infected with DENV2 at a MOI of 2 or mock-infected and incubated at 37 °C, with 5% CO_2_ for 10, 24, and 48 h. Plasma membrane proteins from the above cells were extracted separately, using a BioVision plasma membrane protein extraction kit (BioVision, USA). Approximately 50 µg of the plasma membrane fraction was resolved by 12% SDS-PAGE and transferred onto PVDF membranes, as described above. The membrane containing transferred protein was blocked with blocking buffer, at room temperature, for two hours, and probed with 1:1000 dilution of rabbit polyclonal anti-annexin II antibody, overnight, at 4 °C. The membrane was washed and incubated with 1:1000 dilution of rabbit anti-β-actin antibody (Cell Signaling Tech., Danvers, MA, USA), at room temperature, for two hours. The membrane was washed and incubated with a 1:9000 dilution of goat anti-rabbit HRP-conjugated secondary antibody (Abcam, UK), at room temperature, for two hours, washed, and then developed by ECL. All the washings were performed, three times, with TBS-T, for ten minutes each time. Images were acquired using a Syngene gel/chemi documentation system (Syngene, UK) and densitometry analysis of bands was undertaken using ImageJ software.

### 2.5. Antibody-Mediated Infections Inhibition Assay

Approximately 1 × 10^5^ Vero cells were seeded in 6-well tissue culture plates (Cellstar, Sigma-Aldrich, USA), in an antibiotic-free EMEM and incubated at 37 °C with 5% CO_2_ for 24 h. Filopodia formation was induced, chemically, using 200 ng of bradykinin, and incubated at 37 °C with 5% CO_2_, for 30 min. Vero cells were washed, twice, with PBS and then incubated with various concentrations of rabbit polyclonal anti-annexin II antibody (5 µg, 10 µg, and 20 µg), non-specific control antibody (rabbit polyclonal anti-His tag antibody), or no antibody, at 37 °C, with 5% CO_2_, for one hour. Vero cells were washed, twice, with PBS and infected with DENV2 at a MOI of 2 and incubated at 37 °C, with 5% CO_2_ for one hour. Vero cells were washed, three times, with PBS and fresh virus media (EMEM supplemented with 2% FBS) was added and incubated at 37 °C, with 5% CO_2_, for 30 h. At 30 h, post incubation, the extracellular virus (culture media) was collected separately by centrifugation, at 13,000× *g*, for 30 min. Fresh media was added to the pellet and freeze-thawed for 2–3 cycles and intracellular viral RNA was extracted, using the QIAamp viral RNA kit (Qiagen, Hilden, Germany). The extracellular virus titre was determined by TCID_50_, following the protocol described by Tang et al. [24] and intracellular virus titer was determined using a Liferiver dengue virus general-type real time RT-qPCR Kit (Liferiver Bio-Tech, San Diego, CA, USA), following the manufacturer’s instructions, and as described by Najioullah et al. [25], respectively.

### 2.6. siRNA-mediated Annexin II Gene Silencing

Approximately 2 × 10^5^ Vero cells were plated in 6-well tissue culture plates and maintained in antibiotic-free EMEM. At 60–70% confluence, cells were transfected, using Santa Cruz siRNA transfection kits (Santa Cruz, USA), following the manufacturer’s instruction. Briefly, Vero cells were transfected with various concentrations of annexin II siRNA duplex (Santa Cruz, USA), FITC-conjugated control siRNA or mock-transfected. Annexin II siRNA duplex and control siRNA were separately mixed with siRNA transfection media (sc-36868, Santa Cruz, USA), siRNA transfection reagent (sc-29528, Santa Cruz, USA), and incubated at room temperature, for 40 min. Vero cells were washed, twice, with 2 mL of siRNA transfection media and gently overlaid with siRNA transfection mixture. Vero cells were incubated at 37 °C with 5% CO_2_, for seven hours. At seven hours post-transfection, transfection efficiency was examined in the cells transfected with the FITC-conjugated control siRNA, using a fluorescence microscope (Olympus, Japan). Later 2× normal growth media (EMEM supplemented with 20% FBS and penicillin-streptomycin 10 mL/L (Sigma Aldrich, USA)) was added and incubated, further, at 37 °C with 5% CO_2_ for an additional 17 h, for a total of 24 h. At 24 h post-transfection, total cellular proteins from transfected Vero cells were harvested by rinsing with ice-cold PBS and then homogenized in 300 µL of 2 × SDS sample buffer (65.8% mM Tris-HCl pH 6.8, 26.3% (*w*/*v*) glycerols, 2.1% SDS, and 0.01% bromophenol blue). The plate was gently rocked and the cells were pipetted up and down. The cells were then sonicated on ice with an ultrasonicator XL-2000 series (MisoniX, Farmingdale, NY, USA) at 3–6 W output, for 2–3 min. Approximately 50 µg of total cellular proteins were resolved by the 12% SDS-PAGE. Proteins were transferred to a PVDF membrane, as described above, and the membrane containing the transferred proteins was incubated with a 1:1000 dilution of rabbit polyclonal anti-annexin II antibody (Abcam, UK), at 4 °C overnight. The membrane was washed and incubated with 1:1000 dilution of rabbit anti-β-actin antibody (Cell Signaling Tech., USA), at room temperature, for two hours. The membrane was washed and incubated with a 1:9000 dilution of goat anti-rabbit HRP-conjugated secondary antibody (Abcam, UK), at room temperature, for two hours, washed, and then developed by ECL and images acquired, as described previously. All washings were performed, three times, with TBS-T for ten minutes each. Protein concentration was determined, as described above.

### 2.7. siRNA Transfection and Dengue Virus Serotype 2 Infection

Vero cells were either transfected with 60 pmol of annexin II siRNA duplex, control siRNA or mock-transfected, as described above. At 24 h post-transfection, the transfected Vero cells were washed, twice, with PBS and infected with DENV2 at a MOI 2, and incubated at 37 °C, with 5% CO_2_, for approximately one and a half virus replication cycles (30 h) [26]. At 30 h post-incubation, the extracellular and intracellular virus was collected, processed, and the virus titer was determined, as described above.

### 2.8. Statistical Tests

All statistical tests were performed using the GraphPad Prism program version 6.01 for Windows (San Diego, CA, USA), using either Student’s *t*-test or ANOVA. *p* values less than 0.05 were used for statistical significance.

## 3. Results

### 3.1. Identification of Dengue Virus Serotype 2 Binding Protein on Filopodia

**Figure 1:**
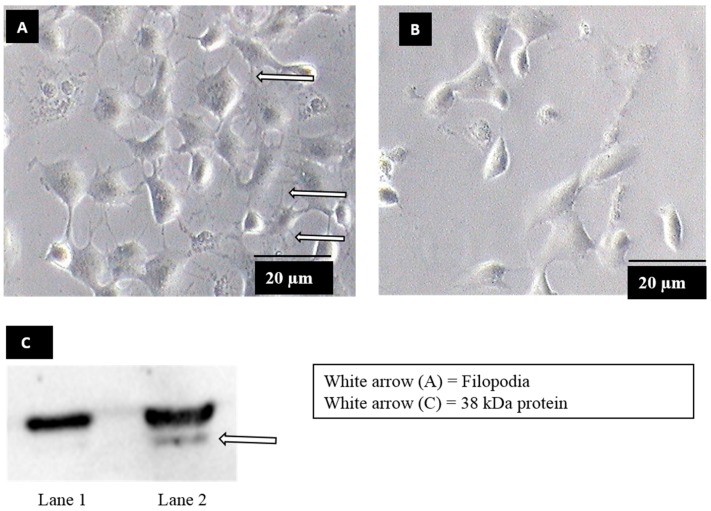
Production of filopodia in dengue virus serotype (DENV) infection and virus overlay protein binding assay (VOPBA) analysis. (**A**) Vero cells were exposed to DENV2 at a multiplicity of infection (MOI) 5, for 30 min. The arrows showed filopodia at the periphery of DENV exposed cells. (**B**) Mock exposed Vero cells showed no filopodia formation. (**C**) A blot of VOPBA analysis of DENV binding to plasma membrane protein extracted from mock-induced (lane 1) and filopodia-induced (Vero cells treated with 200 ng of bradykinin) cells (lane 2). The arrow showed an approximately 38 kDa protein (lane 2) was identified to segregate with DENV2 binding on bradykinin-induced cells.

Filopodia formation was observed when Vero cells were exposed to dengue virus serotype 2 (DENV2) (Figure 1A), whereas no filopodia formation was observed in mock-exposed Vero cells (Figure 1B). In order to identify the molecule on Vero cells upon filopodia formation, bradykinin, a chemical which induces filopodia formation via similar mechanisms to DENV2 [19,27,28] was used. Upon bradykinin treatment, plasma membrane proteins were extracted and subjected to a virus overlay protein binding assay (VOPBA) analysis. DENV2 was shown to bind to an approximately 38 kDa protein, in the plasma membrane fraction of Vero cells treated with bradykinin (filopodia-induction) (Figure 1C, lane 2). The binding was not found in mock-treated Vero cells (mock-induction) (Figure 1C, lane 1). The corresponding band was excised from the parallel Coomassie-blue-stained gels and analyzed by LC-MS/MS. Annexin II was identified on the basis of the spectra generated using the Mascot sequence matching software (Table 1).

### 3.2. Detection of Annexin II upon Dengue Virus Serotype 2 Exposure

**Figure 2:**
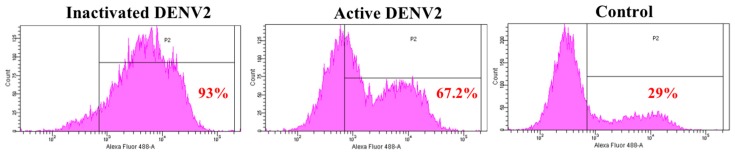
Detection of annexin II on the Vero cells. Vero cells were either exposed with inactivated DENV2 or MOI 2 of DENV2, or mock-exposed for 30 min and 37 °C. Vero cells were fixed and stained with anti-annexin II, followed by Alexa Fluor^®^ 488-conjugated donkey anti-rabbit IgG. The Vero cells were analyzed using the BD FACS Canto II flow cytometer and BD FACSDiva analysis software. Percentage of annexin II positive cells in P1 region is in red.

Annexin II is a pleiotropic cytosolic protein which is known to translocate to the external leaflet of the plasma membrane, in response to certain stimuli [29]. We further examined the expression of annexin II on Vero cells surface, upon DENV2 exposure in flow cytometry analyses. The results showed 93% and 67% of the Vero cells, expressed annexin II on the surface, when exposed to inactivated and active DENV2, respectively, whereas only 29% of Vero cells expressed annexin II on the surface in the control (Figure 2).

### 3.3. Dengue Virus Serotype 2 Interactions with Annexin II

**Figure 3:**
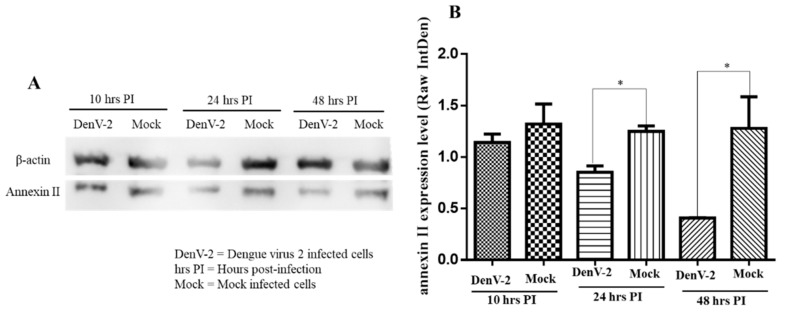
Expression of annexin II in plasma membrane fraction upon DENV2 infection. Vero cells were infected with DENV2 at MOI 2 or mock-infected and incubated for 10, 24, and 48 h. Plasma membrane was extracted, resolved by 12% SDS-PAGE, transferred onto the polyvinylidene difluoride (PVDF) membrane, and developed by electrochemiluminescence (ECL). (**A**) Immunoblots of annexin II expression levels at 10, 24, and 48 h post-infection with DENV2. (**B**) Annexin II band densitometry analysis from panel A. There were significant differences in the annexin II levels between the DENV2-infected and mock-infected Vero cells (* indicates *p* < 0.05) at 24 and 48 h post-infection, using the student t-test. Band densitometry analyses were undertaken using the ImageJ software, after normalization with β–actin (loading control). The value of the raw integrated density was plotted from the three independent experiments and the error bars represent the standard error of the means.

Therefore, western blot analysis was used to determine the responses by assessing the level of annexin II expression in the plasma membrane and the roles of annexin II during DENV2 infection. The amount of annexin II in the plasma membrane fraction was lower, at 24 and 48 h post-infection, compared to the mock-infected cells (*p* < 0.05) whereas no significant difference in annexin II levels was noted at ten hours post-infection (Figure 3A,B).

**Figure 4:**
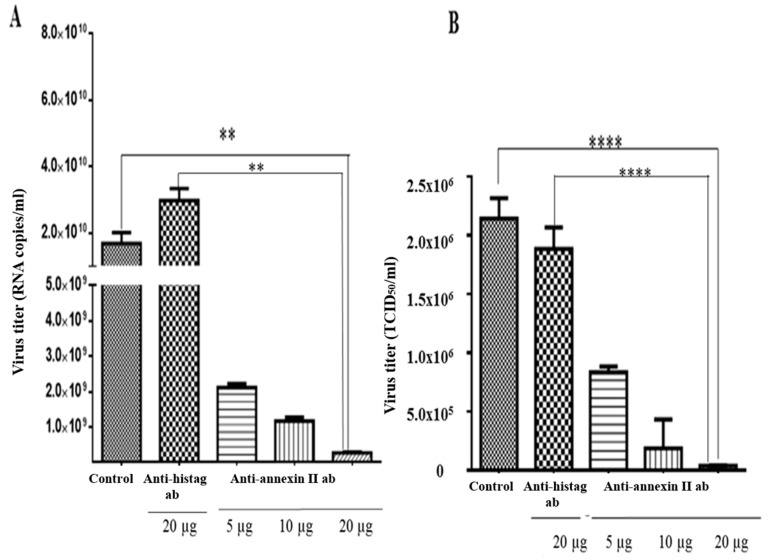
Antibody-mediated DENV2 infection inhibition assay. Vero cells were either treated with various concentrations of rabbit polyclonal anti-annexin II antibody, 20 µg of rabbit polyclonal anti-histag antibody or no antibody (control), for 1 h, prior to DENV2 adsorption. Infections were allowed for 30 h. (**A**) Intracellular DENV2 titer was quantified with Liferiver DENV general-type real time RT-PCR Kit. (**B**) Extracellular DENV2 titer was determined by TCID_50_. The experiment was independently repeated in triplicates. Statistically significant reduction in virus infection and output were observed in the anti-annexin-II-treated Vero cells, compared to the control-antibody-treated and no-antibody-treated Vero cells (** indicates *p* < 0.01, **** indicates *p* < 0.0001), as determined by one-way ANOVA analysis; ab—antibody.

An antibody-mediated infection inhibition assay was performed on the Vero cells, which were pre-incubated with various concentrations of anti-annexin II antibody, prior to the DENV2 infection. Intracellular and extracellular virus titres were determined by RT-qPCR and TCID_50_, respectively. There was a significant reduction in the intracellular levels of the DENV2, in the Vero cells that were pre-incubated with anti-annexin II antibody, compared to the Vero cells that were not exposed to antibody or control-antibody-treated (anti-histag antibody) cells. The greatest inhibition was observed at the highest amount (20 µg) of antibody used in this study (*p* < 0.01) (Figure 4A). Similarly, extracellular DENV2 levels (in culture media) were significantly reduced in a dose-dependent manner in the Vero cells that were pre-incubated with anti-annexin II antibody, compared to the Vero cells that were not exposed to any antibody or the control-antibody-treated (anti-histag antibody) cells (*p* < 0.0001) (Figure 4B).

**Figure 5:**
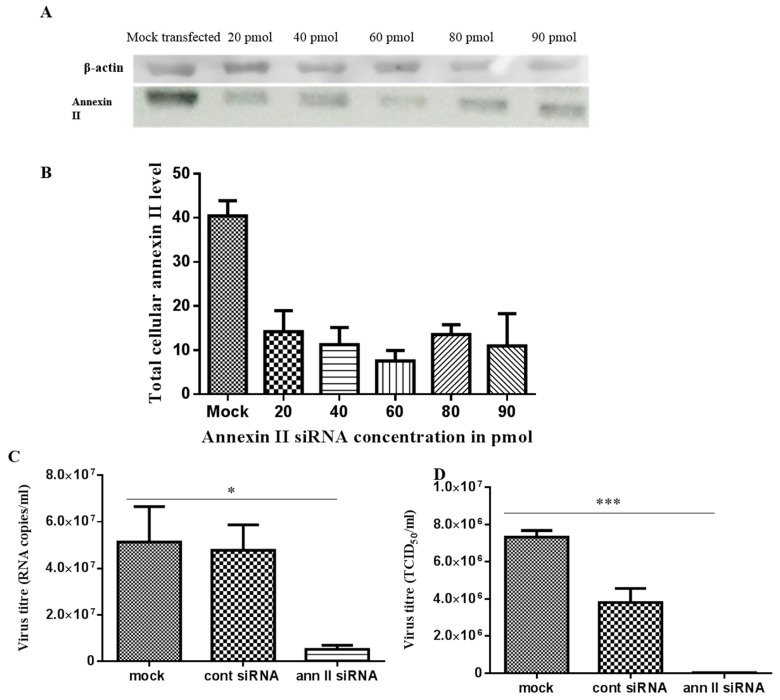
siRNA-mediated annexin II gene silencing and DENV2 infection on annexin II knockdown cells. Vero cells were either transfected with different concentrations of annexin II siRNA duplex, FITC-conjugated control siRNA or mock transfected. Total cellular protein was extracted at 24 h post-transfection and annexin II expression level was detected with anti-annexin II. (**A**) Immunoblots of annexin II expression level transfected with siRNAs. (**B**) Densitometry analysis of annexin II bands from panel “A”. Band densitometry analyses were undertaken using ImageJ software, after normalization with β–actin (loading control). Vero cells were either transfected with 60 pmol of annexin II siRNA duplex, control siRNA, or mock-transfected. The transfected cells were infected with DENV2 at MOI 2 and incubated for 30 h. (**C**) Intracellular DENV2 levels was quantified with Liferiver DENV general-type real time RT-PCR Kit. (**D**) Extracellular virus level was determined by TCID_50_. The experiment was independently repeated in triplicates. There were statistically significant reductions in DENV2 infection and virus output in annexin II knockdown cells, compared to the control siRNA-transfected and mock-transfected cells (* indicates *p* < 0.05, *** indicates *p* < 0.001), as determined by one-way ANOVA. Mock—mock transfected cells. Cont siRNA—control siRNA transfected cells. Ann. II siRNA—annexin II siRNA transfected cells.

In annexin II gene knockdown assays, 60 pmol of annexin II siRNA duplex presented the highest gene knockdown effect, based on immunodetection of annexin II, at 24 h post-transfection (Figure 5A,B). This condition was used to investigate the effect of the annexin II gene knockdown on the production of DENV2. Transfected cells were infected with DENV2, at a multiplicity of infection (MOI) of 2 and incubated for 30 h (approximately 1.5 virus replication cycles). Significant reductions in both intracellular (*p* < 0.05) and extracellular (*p* < 0.001) DENV2 levels were observed in annexin II siRNA-transfected cells, compared to control siRNA-transfected and mock-transfected cells (through ANOVA analysis) (Figure 5C,D).

## 4. Discussion

Envelope viruses have been reported to induce cytoskeletal rearrangement and reorganization which results in filopodia formation, a strategy that results in the confinement of the virus to the cell surface and subsequently delivers them to the internalization receptor [16,30,31]. Murine leukemia virus has been reported to induce actin rearrangement and filopodia formation for viral “surfing” along the formed filopodia, in order to reach the internalization receptor for cellular entry [32]. Furthermore, increased virus uptake has been reported in cells which form filopodia, compared to control cells in which filopodia formation is inhibited in an in vitro experiment [33]. Actin reorganization observed upon cell exposure to certain viruses is a strategy utilized by the virus to hijack cell physiological events for infections. Actin rearrangement, which subsequently results in filopodia formation at the cell periphery, has been reported to prepare cells for enhanced infection [16,34]. However, virus-interacting proteins on these structures, which interact with the virus for increased uptake, have never been reported.

DENV2 has also been reported to induce filopodia formation in HMEC-1 cells and, therefore, blocking of filopodia formation has been observed to inhibit DENV2 infection of these cells [19]. Although the role of filopodia in the confinement of virus to endocytic centers, as well as the delivery of virus to an internalization receptor, has been reported [16,35], the molecular mechanisms of virus-interacting proteins, during filopodia formation, has not been fully elucidated. The present study reports the finding of annexin II, a pleiotropic calcium-dependent phospholipid binding protein, as a DENV2 interacting protein on the plasma membrane, which might get translocated, upon filopodia formation on Vero cells.

Annexin II is an approximately 38 kDa pleiotropic protein that has various functions, for example, endocytosis, fibrinolysis, ion channel formation, and cell matrix interactions [29]. Annexin II is translocated from the cytoplasm to external leaflets of the plasma membrane, upon stimulation [36,37]. In the present study, higher annexin II expression on Vero cells was detected when exposed to DENV2. It is interesting to note that the number of annexin II positive Vero cells was higher when an inactivated virus was used, compared to an active virus. We further investigated the expression level of annexin II, upon DENV2 infection. It was noticed that the amount of annexin II in the plasma membrane fraction was found to be reduced as the infection progressed. Taken together, the above observations might explain how the extracellular translocation of annexin II is stimulated during early DENV2 infection, followed by binding of DENV2 with annexin II, and co-endocytosis during internalization, which reduces the amount of annexin II on the plasma membrane. This was not seen when inactivated DENV2 was used. Involvement of annexin II as an internalization molecule and in enhancing infection, has been reported for human papilloma virus 16 (HPV-16) and cytomegalovirus. HPV-16 induces annexin II translocation to the external leaflets of the plasma membrane in human keratinocytes and this subsequently results in annexin II translocation for virus binding and internalization, whereas, during cytomegalovirus infection, virus inoculation at higher temperatures, increases virus association with annexin II and enhances cytomegalovirus infection [37,38]. Another plausible reason why the level of annexin II in the plasma membrane fraction is reduced when infection progresses, is to prevent super-infection, ensure the establishment of successful infections, and to inhibit interference of the receptor molecules, during virus maturation, assembly, and budding [39]. This was seen in dengue virus, where down-regulation of a 37/67 kDa higher-affinity laminin receptor was reported in HepG2 cells, upon infection, and down-regulation of the receptor protein ATP synthase B subunits in C6/36 cells infected with chikungunya virus [10,40]. The function of annexin II in the present study was proposed to be one of the cellular factor for the dengue virus, because a significant reduction in DENV2 infection and production were demonstrated in antibody-mediated inhibition and gene silencing, respectively. Annexin II has been shown to serve as a receptor molecule in the HPV-16 infection, for binding and internalization [37], and expression of annexin II on the surface of human embryonic rhabdomyosarcoma (RD) cells was observed to enhance enterovirus 71 (EV71) infections [41]. Furthermore, annexin II is reported to play an important role in HIV assembly in monocyte-derived macrophages (MDM) [42]. Besides functioning as a virus receptor, annexin II is also reported to be involved in the recruitment of Hepatitis C virus NS3/NS4A to the lipid raft microdomain, to initiate a viral replication complex in hepatocellular carcinoma cell lines (Huh7.5) [43]. Annexin II also plays a significant role in the pathogenesis of influenza virus (IFV), where annexin II is incorporated into the IFV envelope, during virus budding from infected cells, and annexin II mediates plasminogen conversion to plasmin, which promotes viral replication, invasiveness, and infectiveness [44].

## 5. Conclusions

Significant reductions in DENV2 infection in antibody-mediated inhibition of infection assay and reduction of DENV2 production in gene silencing assay, using siRNA for annexin II suggests that annexin II might serve as a host cell factor involved in DENV2 infection of Vero cells. The findings of this study could serve as a door-opener to further analyze the translocation signaling pathway of annexin II, upon a DENV2 infection, and this might become a target for the design and development of a potent anti-dengue virus vaccine.

## Figures and Tables

**Table 1:** Summary of Mascot searches results from peaks generated in MS/MS analysis.

S/N	Ref. XP	Mass	Score	Matches	Sequence	emPAI	Protein Identity	Species
1	V9HW65	38552	2672	125 (72)	28 (21)	18.63	Human annexin II	*Homo sapiens*
	A0A096NM87	38580	2672	125 (72)	28 (21)	18.63	Papan annexin II	*Papio anubis*
	X008014586.1	38580	2672	125 (72)	28 (21)	18.63	Annexin II	*Cholocebus sabaeus*
2	A0A096NCGO	35959	2085	82 (52)	21 (16)	31.54	Papan Glyceraldehyde-3-phosphate dehydrogenase	*Papio anubus*
3	F6ZXWS	31457	2076	85 (51)	20 (15)	58.60	Glyceraldehyde-3-phosphate dehydrogenase	*Mecaca malutta*
4	A0A0D9SDK6	38721	1829	77 (49)	15 (10)	2.65	Annexin II	*Chlorocebus sabaeus*
